# Feasibility of 3D Reconstruction of Neural Morphology Using Expansion Microscopy and Barcode-Guided Agglomeration

**DOI:** 10.3389/fncom.2017.00097

**Published:** 2017-10-24

**Authors:** Young-Gyu Yoon, Peilun Dai, Jeremy Wohlwend, Jae-Byum Chang, Adam H. Marblestone, Edward S. Boyden

**Affiliations:** ^1^Department of Electrical Engineering and Computer Science, MIT, Cambridge, MA, United States; ^2^MIT Media Lab, MIT, Cambridge, MA, United States; ^3^Department of Brain and Cognitive Sciences, MIT, Cambridge, MA, United States; ^4^Department of Biomedical Engineering, Sungkyunkwan University, Seoul, South Korea; ^5^Department of Biological Engineering, MIT, Cambridge, MA, United States; ^6^McGovern Institute, MIT, Cambridge, MA, United States

**Keywords:** neural morphology, 3-D reconstruction, expansion microscopy, RNA barcode, convolutional neural network, agglomeration

## Abstract

We here introduce and study the properties, via computer simulation, of a candidate automated approach to algorithmic reconstruction of dense neural morphology, based on simulated data of the kind that would be obtained via two emerging molecular technologies—expansion microscopy (ExM) and *in-situ* molecular barcoding. We utilize a convolutional neural network to detect neuronal boundaries from protein-tagged plasma membrane images obtained via ExM, as well as a subsequent supervoxel-merging pipeline guided by optical readout of information-rich, cell-specific nucleic acid barcodes. We attempt to use conservative imaging and labeling parameters, with the goal of establishing a baseline case that points to the potential feasibility of optical circuit reconstruction, leaving open the possibility of higher-performance labeling technologies and algorithms. We find that, even with these conservative assumptions, an all-optical approach to dense neural morphology reconstruction may be possible via the proposed algorithmic framework. Future work should explore both the design-space of chemical labels and barcodes, as well as algorithms, to ultimately enable routine, high-performance optical circuit reconstruction.

## Introduction

In order to analyze neural circuitry in a systematic manner, three-dimensional reconstruction of neural morphology is essential. Optical microscopy has been a key technology for mapping brain circuits throughout the history of neuroscience (Llinás, 2003; Wilt et al., 2009), but it has been limited to imaging and tracing only a small subset of neurons located within a given brain region (Kasthuri and Lichtman, 2007; Miyasaka et al., 2014; Robles et al., 2014), in large part because of the limited spatial resolution of conventional optical microscopy. While it is possible to image thin axons and dendritic spine necks smaller than the diffraction limit of optical microscopes (Dumitriu et al., 2011), this necessitates sparseness of the neurons labeled (i.e., two objects that are each smaller than the resolution, but farther than the resolution from one another, can be resolved). Since only a subset of the neurons within a region can be imaged and resolved under optical microscopy, only partial maps of brain circuitry have been obtained with light microscopy to date. Adding another dimension—color—to the image, by molecularly giving a random color to each neuron (Livet et al., 2007; Cai et al., 2013), increases the number of neurons that can be resolved (Sümbül et al., 2016), but the number is still much lower than the number of neurons in even a modest sized brain circuit because the color becomes locally ambiguous due to optical blurring (and hence color mixing) and the stochastic nature of the distribution of fluorescent molecules within neurons.

Expansion microscopy (ExM) provides a new approach to nanoscale-resolution optical microscopy optimized for application to large 3D brain circuits (Chen F. et al., 2015): in contrast to earlier methods of magnification, which rely on lenses to optically magnify images of preserved brain circuits, ExM physically magnifies samples directly. ExM fuses two sets of ideas: work going back to the 1980s on embedding preserved biological specimens in hydrogels such as polyacrylamide for imaging purposes (Hausen and Dreyer, 1981; Germroth et al., 1995), and work going back to the 1970s on responsive polymers capable of vastly changing size in response to changes chemical environment (Tanaka et al., 1980). By synthesizing a swellable polyelectrolyte gel throughout a specimen, which binds to key biomolecules or labels of interest, then mechanically homogenizing the specimen and dialyzing it in water, we can—by design, and established with extensive validation—expand brain tissues ~4.5x in linear dimension (in our original protocol), with low distortion (~1–4%). Thus, molecules initially located within a diffraction-limited volume are physically separated to distances great enough to be resolved with conventional microscopes, resulting in an effective resolution equal to the diffraction limit divided by the expansion factor, or ~300 nm/~4.5 ≈ ~60–70 nm. We have more recently shown that expansion can be performed multiple times iteratively on the same sample, for 4.5^2^ ≈ 20x linear expansion, or even potentially 4.5^3^ ≈ 100x linear expansion, for two and three iterations respectively (Chang et al., 2017). For the case of 20x expansion, we have validated the ability to resolve nanoscale structures at a resolution of ~25 nm (Chang et al., 2017); the fundamental resolution limit of ExM, the polymer mesh size, may be far smaller, in the range of 2 nm (O'Connell and Brady, 1976; Kurenkov et al., 2001) for the polymers used—in the ball park of the resolution of electron microscopy. Indeed, in our study on 20x expansion, we estimated that the error added by gelation and expansion was on the order of ~5–10 nm, suggesting that the fundamental resolution of ExM could be quite good if other contributors to error (e.g., the sizes of antibodies or other labels), are minimized in magnitude.

These properties of ExM may make it optimal for *in-situ* readout of nucleic acid “barcodes” that have previously been proposed (Zador et al., 2012; Marblestone et al., 2014) to allow unique identification of single neurons (Lu et al., 2011; Kebschull et al., 2016). A “barcode” is defined here as a sequence of RNA used to uniquely “tag” a particular cell with a physical indicator of its identity. The number of possible nucleic acid barcodes grows exponentially with RNA sequence length: there are 4^N^ possible sequences of length N. For example, a barcode of length 30 has a potential diversity of 4^30^ ~ 10^18^, a number that vastly outstrips the number of neurons in the mouse brain (~10^8^) or even those of the largest mammals. Thus with sufficient diversity, ensured by a sufficiently long barcode sequence length, each neuron in a brain will almost certainly express a unique tag, even when barcodes are generated, or delivered to neurons, at random. Such barcodes could be read out optically in an expanded ExM specimen through the use of fluorescent *in-situ* sequencing (FISSEQ) (Lee et al., 2014; Chen et al., 2017) or fluorescence *in-situ* hybridization (FISH) (Langer-Safer et al., 1982; Chen et al., 2016). The barcodes, when read out *in-situ*, appear as spatially localized fluorescent dots (occupying ~400 nm size post-expansion, or ~20 nm effective size compared to other biological structures under 20x ExM), which blink out a digital string of colors that uniquely encodes the identity of the cell that contains them, during the repeated steps of *in-situ* sequencing.

**Figure 1:**
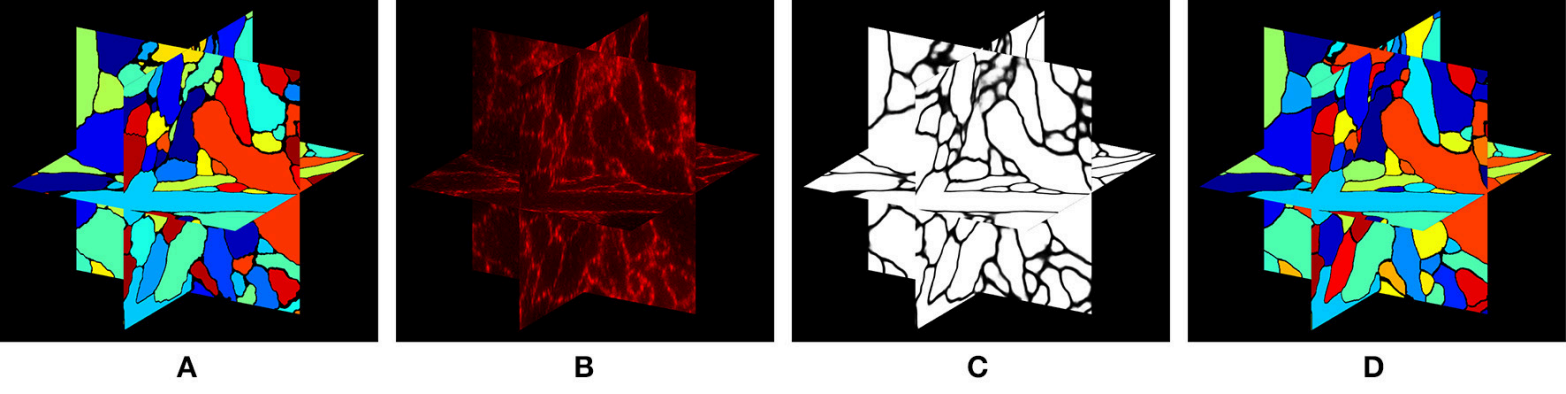
Simulation of ExM volume reconstruction pipeline. **(A)** Ground truth input dataset obtained from fully reconstructed electron microscopic volume. **(B)** Simulated optical ExM image. **(C)** Boundary detection performed on simulated optical ExM image. **(D)** 3D neuron segmentation obtained from simulated optical ExM image.

In this paper, we approach the question of dense 3-D neural morphology reconstruction theoretically, in light of the large-volume 3D optical super-resolution capability enabled by ExM, combined with the molecular identification capabilities supported by optical approaches. We ask: what are the limits of an optical approach in the context of 3-D reconstruction of neural morphology with ExM, and how can we approach those limits through novel chemical tags and analytical protocols that take advantage of ExM, as well as specific algorithms? Since such questions must be answered in the context of the detailed structural properties of neural tissue, we base our analysis on a simulation of ExM imaging which uses electron microscopic volume reconstructions of neural tissue (Figure 1A) as a “ground truth”—our simulation, then, produces images which emulate those which would be generated by ExM in an optical confocal microscope (Figure 1B), on a neural volume with a precisely known structure previously obtained by electron microscopy and analyzed. This approach is illustrated in Figure 1.

In our current study, we take a conservative approach, both in our estimates of the performance of ExM and in our algorithmic design. Indeed, we aim to demonstrate the feasibility of a baseline optical reconstruction approach using already-demonstrated ExM performance in terms of resolution and noise, already-demonstrated neuronal labeling methods compatible with ExM, and straightforward algorithms from the electron microscopy connectomics field. Although the future will likely bring substantial improvements in the performance of all these technology components, our aim here is to demonstrate a baseline case that establishes the basic feasibility of a general class of optical approaches. Specifically, we simulate realistic, often pessimistic, conditions with important informational limitations, e.g., limited fluorophore density, nonzero background fluorescence, limited spatial precision of fluorophore targeting of membranes, and modest expansion factors (see Table 1). Such parameters have been roughly matched to the properties of an ExM protocol with ~20x linear expansion factor that we recently demonstrated experimentally (Chang et al., 2017). We also choose a conservative algorithmic approach, taking one that is similar to that used by the electron microscopy connectomics community, namely boundary detection using a convolutional neural network (ConvNet) (Figure 1C). Subsequent steps of the segmentation pipeline, such as watershed oversegmentation and supervoxel merging, are also performed in a manner analogous to that used in electron microscopy connectomics. We characterized the error rate of this ConvNet-based image segmentation process on ExM data simulated from ground-truth electron microscopy data, and found that it was possible to obtain Rand scores and information theoretic scores above 0.9.

**Table 1:** Simulation parameters.

	**Parameter**	**Value**
Confocal microscopy	Magnification (M) of objective lens	40
	Numerical aperture (NA) of objective lens	1.15
	Physical pixel pitch	4.8 (μm)
	z step	120 (nm)
	Signal-to-noise ratio (read noise; at the brightest pixel)	50–100
	Signal-to-noise ratio (Poisson noise; at the brightest pixel)	7–12
Expansion	Expansion factor	20
Fluorophore	Density (membrane)	4,000–10,000 (puncta/μm^2^)
	Density (cytosol)	2,000–4,000 (puncta/μm^3^)
	Density (background)	1,000–2,000 (puncta/μm^3^)
	Density (barcodes)	5–300 (molecules/μm^3^)
	Localization accuracy [standard deviation (SD)]	20 (nm)
	Size of local clusters (SD)	1–48 (nm)

Taking advantage of the ability of ExM to support multiplexed molecular interrogation, we further studied whether this performance could be enhanced via the use of previously proposed nucleic acid sequencing approaches for cell identity readout based on cell-specific RNA barcodes (Zador et al., 2012; Marblestone et al., 2014; Peikon et al., 2014; Kebschull et al., 2016). We found that such a combined approach could allow higher-accuracy 3D reconstructions of neural volumes, with both Rand scores and information theoretic scores above 0.95, by using cell identity information from barcodes to guide the merging of supervoxels derived from the boundary detector. We studied the performance of this integrated pipeline—which combines machine learning for boundary detection with *in-situ* nucleic acid barcode sequencing for supervoxel merging—as a function of barcode density, and found that barcodes could improve the accuracy of reconstructions with realistic numbers of RNA barcodes per cell, assuming that barcodes could be randomly and uniformly distributed throughout the cytosol (see section Discussion for details on barcode targeting).

In summary, in what follows, we first introduce the ExM simulation approach. We then study the performance of a reconstruction pipeline using simulated 20x ExM data with a protein-based plasma membrane label similar to the membrane-anchored BrainBow constructs used with ExM in Tillberg et al. (2016) and Chang et al. (2017). We then introduce an integrated reconstruction pipeline that incorporates both membrane morphology and barcode readout. The morphology information from the 20x ExM membrane label can be processed in a similar way to how electron microscopy images are processed for 3-D reconstruction of neural circuits (Liu et al., 2012; Nunez-Iglesias et al., 2013): machine learning is employed to obtain a boundary probability map (BPM) from a membrane image, and then the BPM is then processed to find the connected components. The BPM is first over-segmented with a conservative threshold to ensure that there is no false merging of different neurons, and then the resulting segments are merged to obtain the final segmentation. In addition, we propose to read out neuronal identity information via *in-situ* multiplexed readout of barcodes distributed randomly throughout the neuronal cytosol. The barcode identities are then used as an input to the merging step, and we describe how to use barcodes as a means to guide segment mergers. In the discussion, we argue that such a pipeline could extend 3-D reconstruction to much larger volumes by allowing error correction over short- and long-range distances and allow facile integration with *in-situ* readout of cellular and synaptic molecular markers. Thus, our theoretical and computational analysis may contribute to the formulation of novel, useful optical approaches to high-resolution brain mapping.

## Results

### Simulation of expansion microscopy images

Simulation of ExM images was performed by first simulating stochastic fluorescent labeling of a volume of neural tissue and then projecting the tissue onto a 3-D image stack using an optical model of a confocal microscope. We used electron microscopic volume reconstructions of mouse cortex from (Berger et al., 2013; Kasthuri et al., 2015) as an input for the simulation.

**Figure 2:**
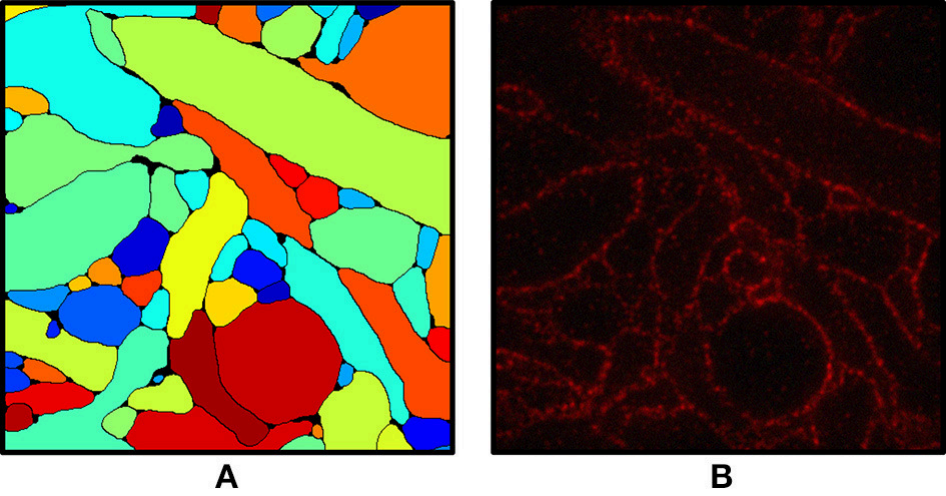
**(A)** Ground truth labeling used as the input for the simulation. **(B)** Simulated ExM image from **(A)**. Fluorophores were randomly assigned to the membrane voxels and non-membrane voxels and the volume was projected onto an image stack by convolving with the 3-D PSF of the microscope, then adding Poisson noise and read-out noise. The field of view is ~3 × 3 μm.

Figure 2 shows an input slice and the corresponding output slice from our simulation. All neuronal membranes were labeled with red fluorophores, with varying labeling density across neurons. As a result, some of the neurons had relatively clear boundaries whereas the boundaries of other neurons were less clear. In addition, due to the discrete nature of fluorophores, they appeared at this magnification (see Chang et al., 2017 for actual data) punctate rather than as continuous contours (Figure 2B). Interestingly, we found that the discernibility of neurons with very low labeling density was improved when their membranes touched the membranes of neighboring neurons.

**Figure 3:**
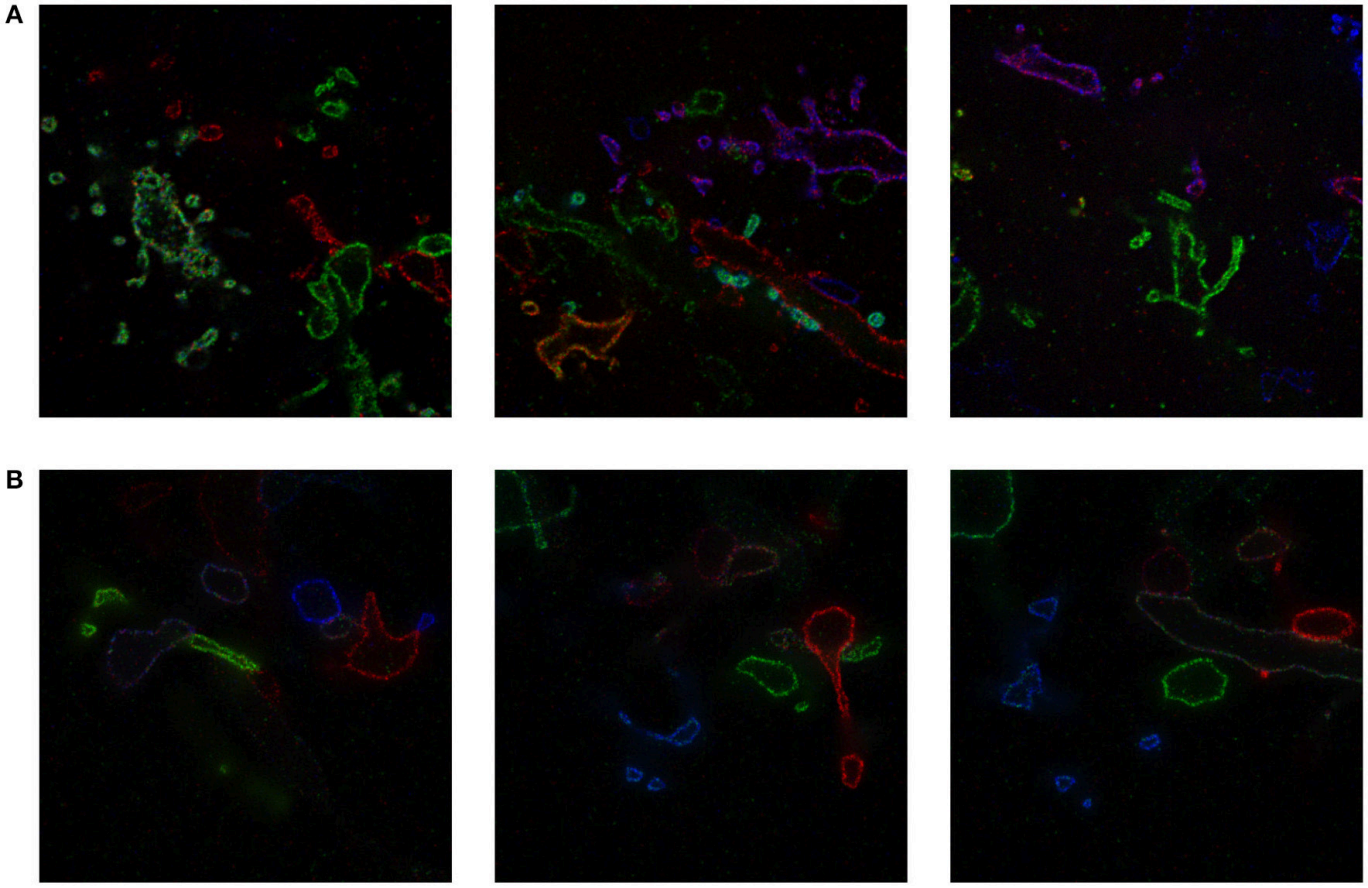
ExM image compared to simulated ExM image. **(A)** Real ExM images of immunostained Emx1-Cre mouse hippocampus with neurons expressing membrane-bound fluorescent proteins (Brainbow 3.0) which are tagged via primary and secondary antibodies bearing a fluorescent label and expanded twenty-fold, followed by a post-expansion fluorescence amplification step. **(B)** Simulated ExM images obtained by labeling only a small set of neurons in multiple colors, intended to match the imaging conditions from **(A)**. The field of view of each image is ~ 6 × 6 μm pre-expansion.

We include a real 20x ExM image side by side with a simulated ExM image to illustrate the realism of our simulation. Figure 3A shows z-slices from a confocal image stack of immunostained Emx1-Cre mouse hippocampus with neurons expressing membrane-bound fluorescent proteins (Brainbow 3.0; Cai et al., 2013) obtained with the 20x ExM protocol and Figure 3B shows z-slices from an image stack simulated from electron microscopy data. The average color for each neuron was randomly selected, and then red, green, and blue fluorophores were randomly assigned to the voxels in a mutually exclusive fashion, to simulate BrainBow.

### Segmentation

#### Boundary detection

**Figure 4:**
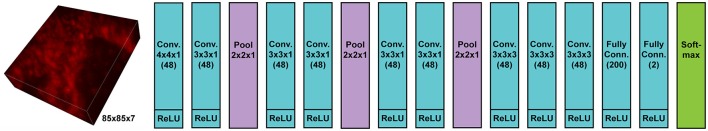
Architecture of 3-D convolutional neural network for boundary detection. The network has nine convolution layers, three max-pooling layers, two fully-connected layers, and a softmax layer. Rectified linear unit (ReLU) was used as the activation function.

**Figure 5:**
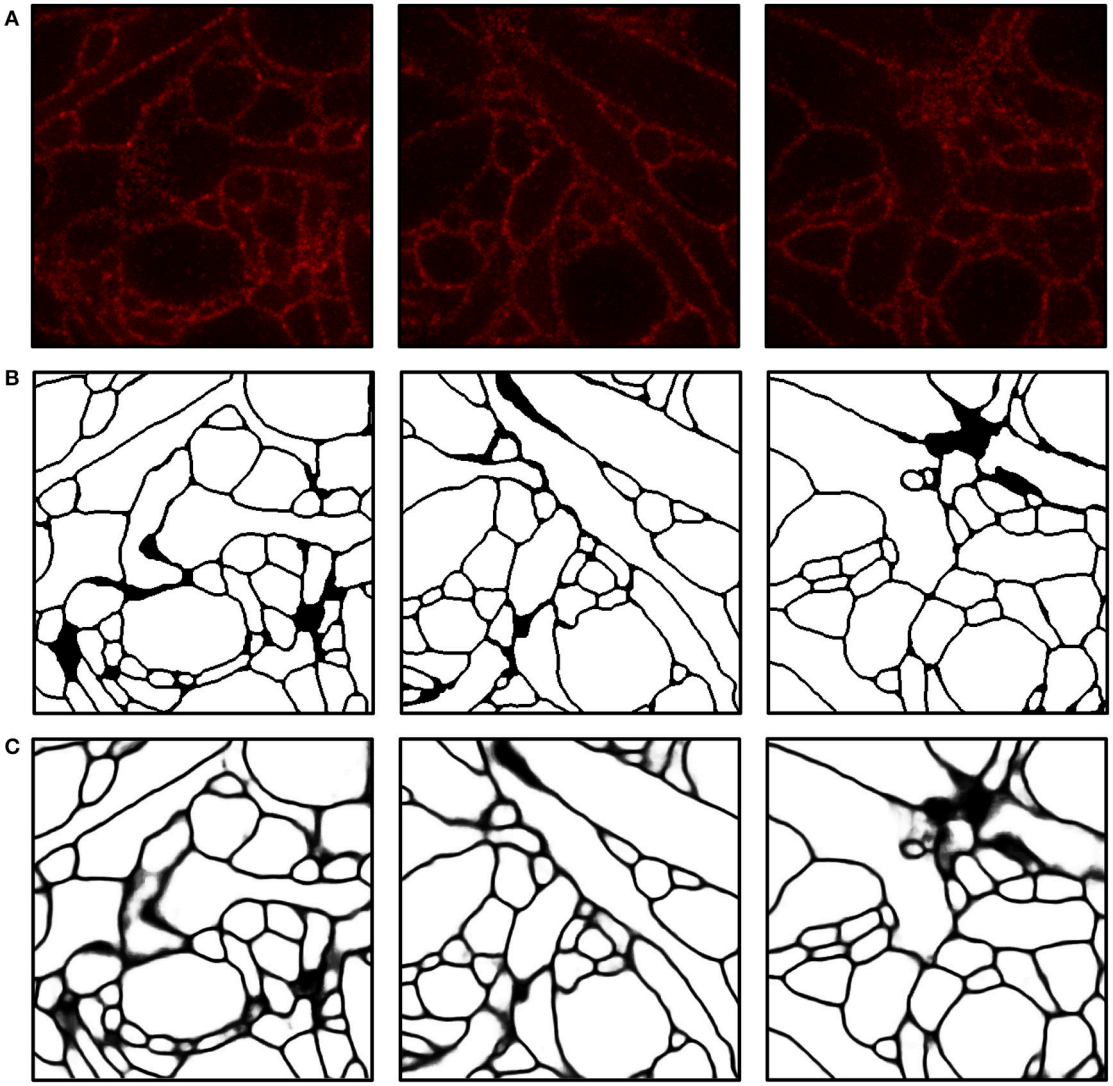
Boundary detection using a convolutional neural network. **(A)** Input images. **(B)** Ground truth boundary map. **(C)** Boundary probability map obtained by combining three outputs from ConvNets and applying a 3-D median filter (see Materials and Methods for details). Boundary probabilities of 1 and 0 are mapped to black and white, respectively. The field of view of each image is 2.4 × 2.4μm.

We implemented and used a 3-D ConvNet for boundary detection by adopting and modifying the neural network design from VD2D3D (Lee et al., 2015); our network architecture is shown in Figure 4. A BPM was obtained as the output of the ConvNet where each pixel in the BPM represents the probability of the pixel being on the boundary of a neuron (Ciresan et al., 2012). As the boundary at certain locations was not clear from a single z-slice, due to the limited number of fluorophores and resolution, using a 3-D ConvNet architecture was important to provide volumetric context to the network. It should be noted that seven z-slices were fed into the 3-D ConvNet instead of just a single slice, as shown in the figure; also, every third slice was picked instead of picking consecutive slices. By sampling every third slice, the amount of redundant information in the slices was reduced and the volumetric context from a larger volume could be provided to the ConvNet. A few input slices to the ConvNet and their corresponding outputs are shown in Figure 5.

#### Over-segmentation and agglomeration

**Figure 6:**
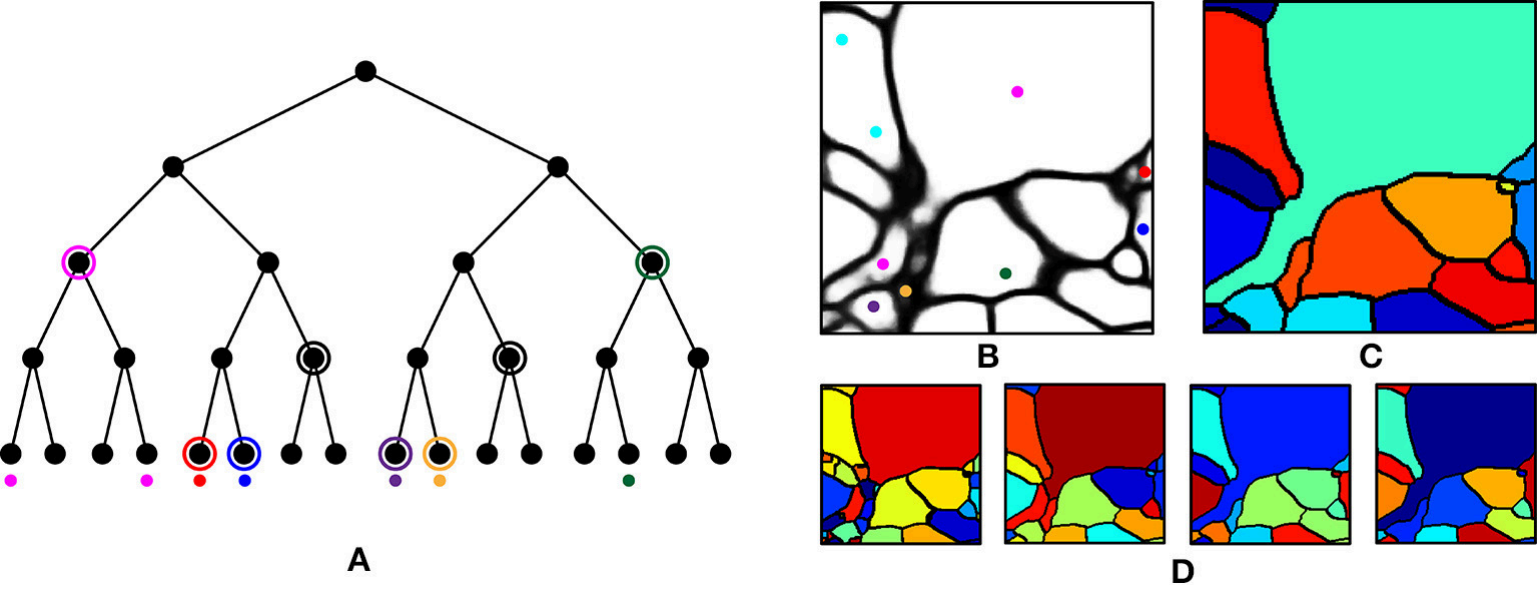
Agglomeration algorithm using barcodes. **(A)** Illustration of our agglomeration algorithm using hierarchical segmentation tree and barcodes. Here, the hierarchical segmentation is obtained by applying GALA to the initial over-segmentation with different threshold values. Colored dots represent barcodes and their colors represent their identity. Highest levels of the nodes are found (circled) that do not contain different barcodes (as would result from a strategy of merging whenever barcodes do not mismatch). **(B)** Barcodes (represented as colored dots) overlaid on the boundary probability map. Each color represents a unique sequence of barcodes. **(C)** Final segmentation obtained by using the barcodes in **(B)** and the hierarchical segmentation tree in **(D)**. **(D)** Four levels of hierarchical segmentation from GALA.

We first performed over-segmentation of the volume by applying a watershed transform to the BPM from the ConvNet and then the resulting segments were merged using the graph-based active learning of agglomeration (GALA) algorithm (Nunez-Iglesias et al., 2013, 2014), an agglomeration algorithm based on supervised learning of a policy that determines whether each pair of adjacent supervoxels should be merged or not. A hierarchical segmentation tree was obtained by applying GALA to the test image with multiple threshold values (Figure 6A), where a higher threshold value provided a result with more merges. This tree can be interpreted as a pool of segmentations in which different neurons are better reconstructed in different levels, i.e., there is no single threshold value that is ideal for all neurons. In other words, each node in the tree serves as a segmentation hypothesis and this hypothesis is either approved or rejected by the barcode consistency within the node (i.e., a node is inconsistent if more than one distinct barcode is found in a single node). Finally, nodes with the same barcode were merged. This procedure is illustrated in Figure 6. It should be noted that this algorithm allowed the agglomeration of segments that were disconnected by mistake, or not even connected in the field of view. This is important for large-scale reconstruction, as it shows how barcodes can prevent “propagation” of local errors and can help support the correction of errors without human intervention.

**Figure 7:**
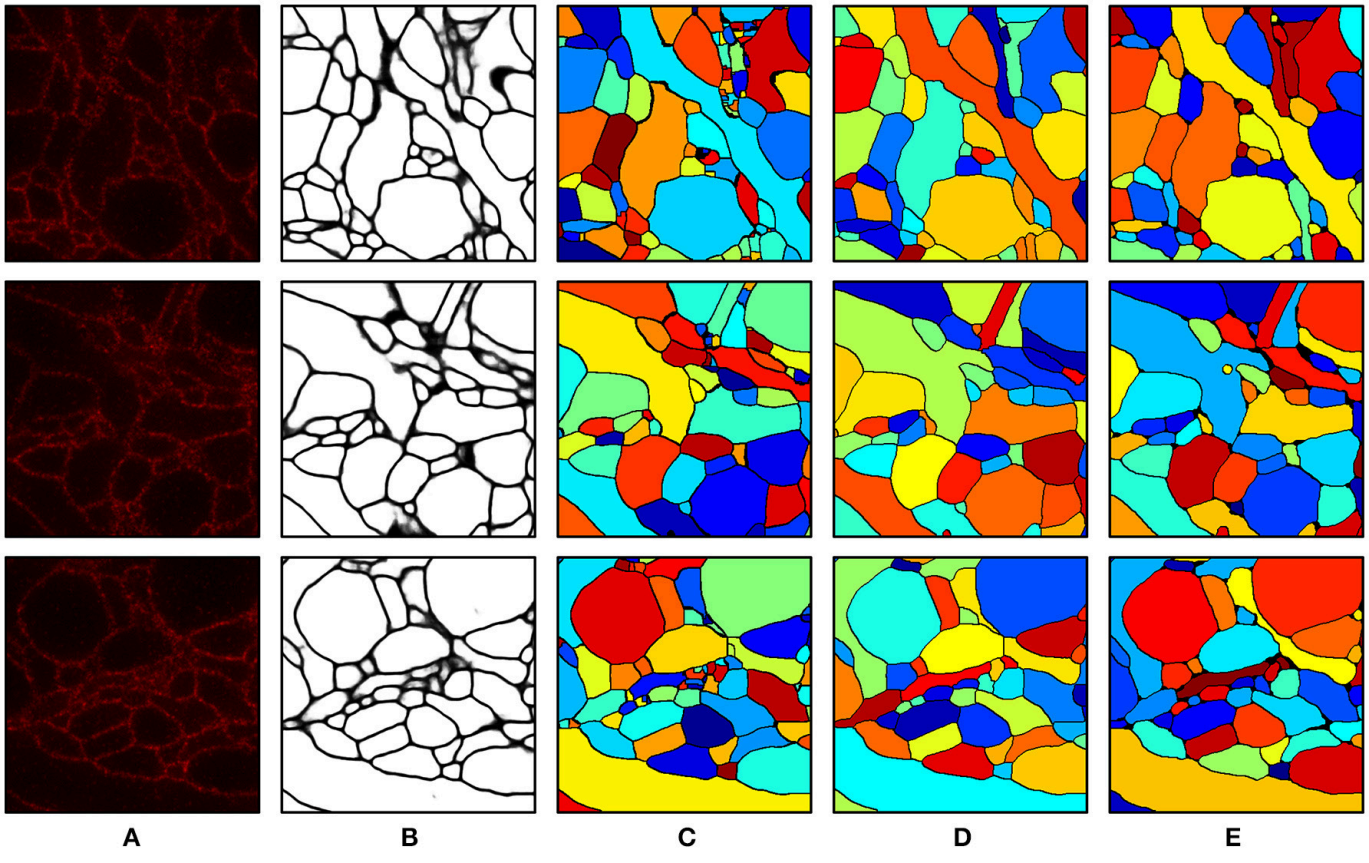
Segmentation results. **(A)** Input image. **(B)** Boundary probability map. **(C)** Initial over-segmentation obtained by applying watershed transformation to the boundary probability map. **(D)** Final segmentation obtained by agglomerating **(C)** using GALA and barcodes. Barcodes with a density of 30 barcodes/μm^3^ were used for the agglomeration. **(E)** Ground truth. The field of view of each image is 2.4 × 2.4 μm.

**Figure 8:**
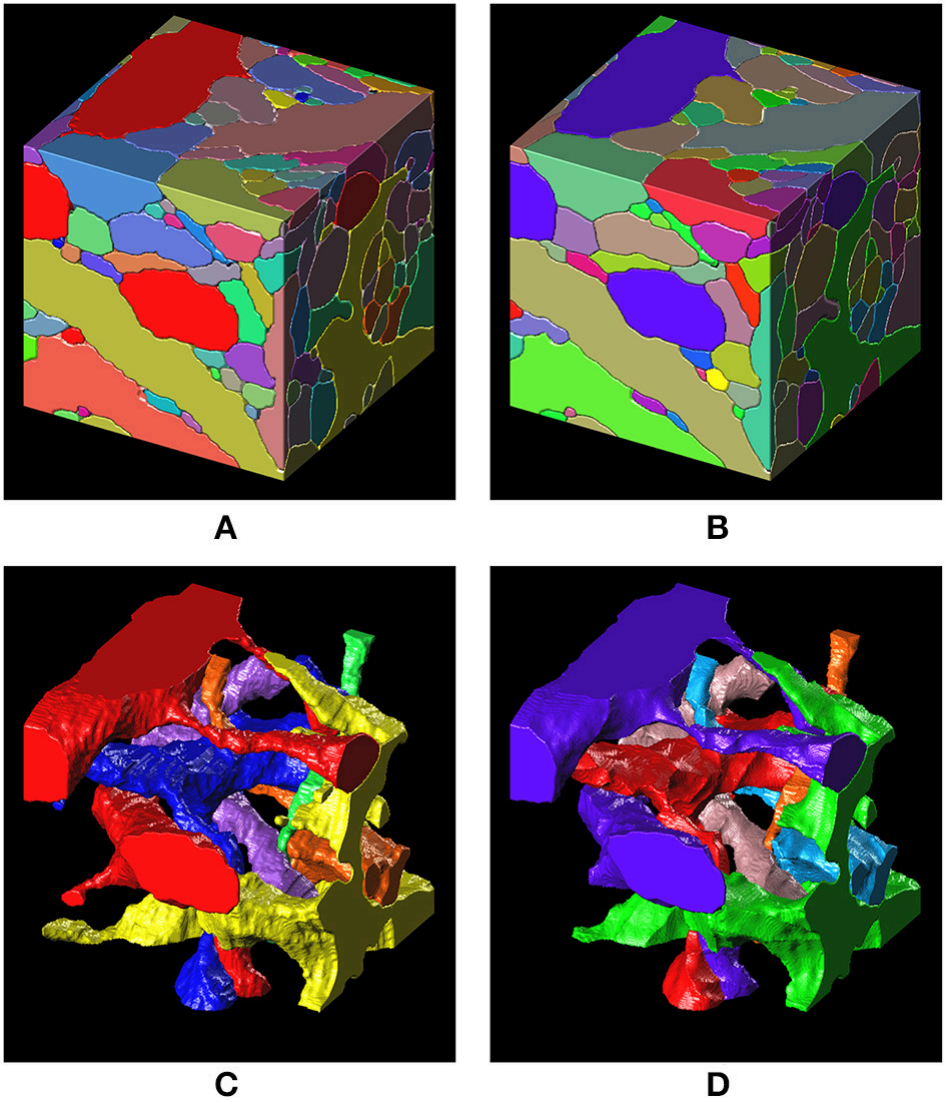
3-D surface renderings of the ground truth and the segmentation result. **(A,C)** 3-D surface renderings of the ground truth input data set. Only six segments are shown in **(C)**. **(B,D)** 3-D surface renderings of the segmentation result obtained with a barcode density of 30 barcodes/μm^3^. Only six segments, that correspond to the segments in **(C)**, are shown in **(D)**.

Intermediate and final segmentation results from our segmentation pipeline are shown in Figure 7 along with the input image and the ground truth; 3-D surface renderings of the ground truth and the final segmentation are shown in Figure 8. An H-minima transform, followed by a watershed transform, yielded a good initial over-segmentation for subsequent agglomeration steps in the sense that both under-segmentation and severe over-segmentation were avoided. Most over-segmentation occurred in thin neurites running parallel to the z-planes, where the ConvNet failed to provide clear boundaries due to the anisotropic resolution (~200 × 200 × 600 nm) of optical confocal microscopy that we assumed in our simulations, although this resolution is better for more modern microscope architectures (see section Discussion).

#### Quantification of segmentation accuracy

For quantitative assessment of segmentation quality, we used F-scores of the Rand index and variation of information (VI) (Arganda-Carreras et al., 2015), which are measures of the similarity between two segmentations. The Rand split score and Rand merge score are defined as follows, where *n*_*ij*_ denotes the number of joint voxels of the i^th^ segment of the predicted segmentation *X* and the j^th^ segment of the ground truth segmentation *Y*, and the Rand F-score is the harmonic mean of the Rand split score and the Rand merge score:

(1) $$\begin{array}{r} V_{split}^{Rand}=\frac{\sum_{ij}n_{ij}^{2}}{\sum_{i}{\left(\sum_{j}n_{ij}\right)}^{2}},\;V_{merge}^{Rand}=\frac{\sum_{ij}n_{ij}^{2}}{\sum_{j}{\left(\sum_{i}n_{ij}\right)}^{2}}. \end{array}$$

Similarly, the information theoretic split score and the information theoretic merge score are defined as follows where I(X,Y), H(X), and H(Y) denote the mutual information between X and Y, the entropy of X, and the entropy of Y, respectively, and their harmonic mean is the information theoretic score:

(2) $$\begin{array}{r} V_{split}^{VI}=\frac{\text{ I}\left(\text{X},\text{Y}\right)}{H\left(X\right)},\;V_{merge}^{VI}=\frac{\text{ I}\left(\text{X},\text{Y}\right)}{H\left(Y\right)}. \end{array}$$

**Figure 9:**
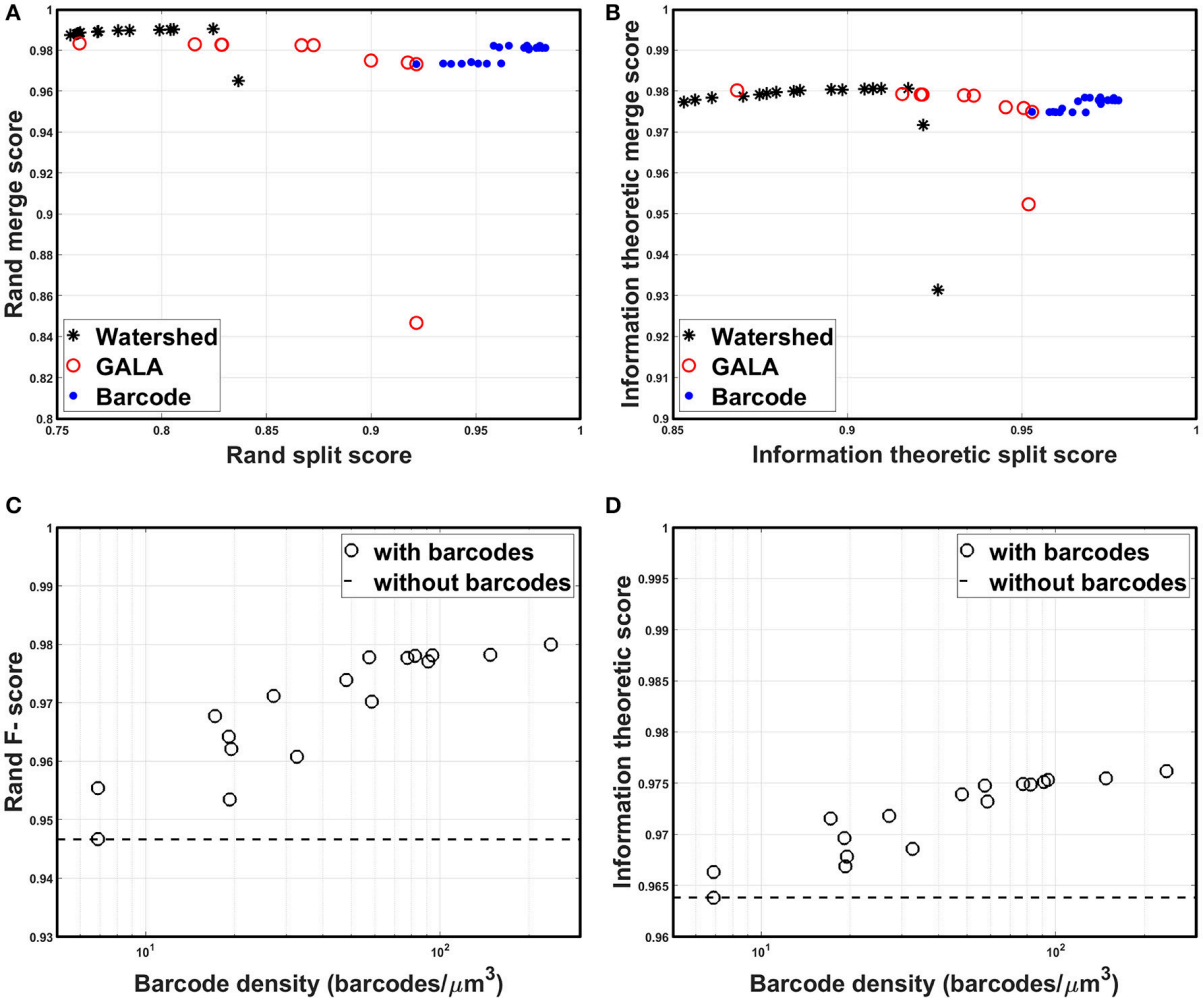
Segmentation accuracy. **(A)** Rand split score vs. Rand merge score at each step of the segmentation pipeline: direct watershed segmentation, GALA, barcode-based agglomeration. Barcodes with pre-expansion densities of 5–300 barcodes/μm^3^ were used for the agglomeration. **(B)** Information theoretic split score vs. information theoretic merge score at each step of the segmentation pipeline: direct watershed segmentation, GALA, barcode-based agglomeration. Barcodes with pre-expansion densities of 5–300 barcodes/μm^3^ were used for the agglomeration. **(C)** Rand F-scores as a function of barcode density. **(D)** Information theoretic scores as a function of barcode density.

All scores are bounded between zero and one, by construction, with higher scores indicating more accurate segmentation. Foreground-restricted F-scores (i.e., F-scores computed only for voxels that are non-boundary in the ground truth data) were evaluated, as they were less sensitive to small border variations (Arganda-Carreras et al., 2015); the results are summarized in Figure 9. To quantify the segmentation quality achievable by the watershed transformation, the precision-recall curve, or the split score vs. merge score curve, was obtained by applying the H-minima transform with various threshold values as in Arganda-Carreras et al. (2015); Lee et al. (2015), and Nunez-Iglesias et al. (2014). To create a precision-recall curve for segmentation quality when barcodes were not used, each level of the hierarchical segmentation tree, which was obtained using GALA with a specific threshold value, was compared to the ground truth to measure its score. From Figures 9A,B, it can be seen that the split scores were significantly improved at each step of the pipeline (e.g., as barcodes are added), without compromising the merge score. The segmentation quality as a function of the barcode density was also measured and the results are shown in Figures 9C,D. For each trial, we generated a new set of randomly distributed barcodes with a given density for the otherwise identical data set and used it for the agglomeration step. The result was different for each trial even with the same density of barcodes, as in some cases barcodes were located at critical locations and corrected major errors, whereas in some cases they were not. With the higher barcode density, it was more likely to have barcodes in such critical locations, and thus, scores tended to increase as the barcode density was increased. The F-scores did not asymptotically reach unity as the barcode density was increased, as the quality of the initial over-segmentation imposed an upper bound on the accuracy of the agglomeration result. To solve this problem, future algorithms could explore methods to incorporate barcode information earlier in the segmentation pipeline, e.g., during the initial generation of the over-segmentation, or to use improved tags, e.g., with higher localization accuracy, to improve the quality of the initial BPM and over-segmentation (see section Discussion for details). Concretely, barcodes with pre-expansion densities of 5–300 barcodes/μm^3^ (the density is ~8,000-fold lower following 20x expansion) led to a significant improvement in segmentation quality scores (Figures 9A,B). These are high densities, but it should be noted that RNA readout with a density of 20 molecules/μm^3^ has been previously demonstrated using multiplexed RNA fluorescent *in-situ* hybridization (FISH) (Chen K. H. et al., 2015), even without the benefit of ExM. We expect that these kinds of barcode densities could be achievable by combining highly expressed RNA barcodes with an efficient readout step, e.g., leveraging the high efficiencies of single-molecule RNA FISH, although the details of the subcellular targeting and distribution of barcodes will be a key question for most barcoding approaches, e.g., whether barcodes are excluded from certain regions under a given targeting scheme. Moreover, other schemes for incorporating barcode information may place less stringent requirements on barcode density (see Discussion for details).

## Discussion

We have shown via simulations that, using demonstrated methods of 20x physical expansion, methods of labeling the neuronal cell membrane with fluorescent dyes, and standard confocal microscopes, it would be possible to use convolutional neural networks for boundary detection, and graph-based super-voxel agglomeration, to obtain dense 3-D reconstruction of neural morphologies in brain circuits. Our work thus opens up the possibility of an optical approach to dense reconstruction of neural circuitry, using a combination of demonstrated methodologies. As discussed in the introduction, an optical approach could be valuable for brain mapping by allowing dense 3-D reconstruction of neural circuitry, by enabling multi-color labels such as antibody labels for distinct synaptic sub-types, by enabling integration with *in-situ* transcriptomic and proteomic measurements, and by allowing the use of simple and widely available optical hardware such as confocal microscopes.

We also showed how the use of information-rich cell-identifying RNA barcodes could be incorporated into standard pipelines for image segmentation, by using them to guide super-voxel mergers to improve the accuracy of local reconstruction. We have been deliberately conservative here, focusing on a baseline implementation of an optical reconstruction pipeline, and making no attempt to construct a truly optimal pipeline, nor to push the performance of ExM beyond what has been demonstrated, aside from assuming, in the later sections of the paper, the possibility of high-accuracy barcode readout *in-situ*. There are therefore many directions that could be taken which could lead to major improvements, or to more powerful optical pipelines.

Our simulation study focused on a case where the membranes of all neurons were labeled in a single color for dense reconstruction of neural circuits, but there can be many other scenarios thanks to a diversity of fluorescent labeling strategies. For example, antibody labeling synaptic proteins would allow us to detect synapses directly, and hence, would be a relatively simple, yet powerful, addition to membrane labeling. Pan-neuronal BrainBow could also be useful as it could provide boundary information and cell identity information at the same time (although of course BrainBow is not able to make as many distinct colors as can be created through RNA barcoding, so some neuron-neuron contacts could be ambiguous to segment should they be the same color). Both boundary detection and agglomeration could benefit from such color information, as a multi-color ConvNet is likely to perform better than a single-color counterpart, and agglomeration of colored segments is likely to be an easier task (i.e., BrainBow can serve as an analog form of barcoding that has less information per fluorophore but is spatially more abundant than RNA barcodes).

We have demonstrated one way to incorporate cell-identifying digital barcodes into the segmentation pipeline, by using barcodes as indicators for agglomeration of supervoxels, but the potential of barcodes in the context of brain mapping is much wider. For example, they could be used to guide reinforcement learning of the agglomeration algorithm in place of human annotation, since “correctness” of the merges could be inferred from the barcode consistency at each step of the agglomeration. Barcodes could also be used in a context similar to the “skeleton trace” (Berning et al., 2015), as it directly provides the ground truth for a set of points down the long axis of each neurite. One recent segmentation algorithm (Januszewski et al., 2016) performs image segmentation by “flood-filling” from an initial seed point using a learned flood-filling policy; barcodes could be directly used as seed points as well as a set of points that could be used to check for flood-fills that were overaggressive or that fell short.

Devising new algorithms from scratch specifically to exploit barcode information optimally may prove a particularly fruitful direction for future research. In particular, a framework that can deal with erroneous barcode readout will be of great utility. Our algorithm assumed that barcodes can be read out without errors for simplicity, but in practice barcodes will have a nonzero error rate determined by sequence errors, the chemical specificity of fluorescent probing, and the optical noise level. Such sequence errors could be handled by many different approaches. For example, maximum a posteriori estimation could be used to perform error correction by using a known pool of barcodes. With such an approach, barcode error rate, analogous to the symbol error rate in digital communication, could be made arbitrarily low if the RNA sequence lengths greatly exceeded log_4_(number of neurons in the brain). Instead of digitizing and error-correcting each barcode, unsupervised learning could also be employed to classify all barcodes in the volume. For instance, the unsupervised clustering algorithm DBSCAN (Ester et al., 1996) could be performed, with the data points defined by the spatial locations of barcodes and their color space intensity values during sequencing, to identify the entire pool of barcodes as well as the identity of each barcode.

One assumption utilized here is that barcodes are distributed throughout the entire cell, including fine processes such as spines and presynaptic terminals. While fusion of barcodes to synapse-targeting motifs has been shown to lead to their dispersal throughout long axons (at least for some cell types), it remains to be demonstrated whether barcodes can be made completely cell filling (Kebschull et al., 2016), e.g., including spine necks or other fine processes, and whether these molecular trafficking pathways are conserved across all neuron types. We have chosen in the current study not to make use of barcodes located near to the plasma membrane, because the finite localization accuracy of microscopy can contribute to a kind of error wherein barcodes near the cell boundary are mistakenly found on the other side of the boundary. Indeed, here we chose to ignore barcodes close to boundaries (but this approach is sub-optimal as it makes barcodes in fine processes less likely to be analyzed). A future version of the segmentation algorithm would benefit from a framework that incorporates barcode error-correction at the sequence level, as well as a rating of the confidence with which each barcode should be trusted, as determined by its spatial proximity to the nearest boundary.

Future molecular and algorithmic innovations could directly confront these limitations concerning achievable barcode density, targeting accuracy, and localization uniformity. For example, barcodes could be deliberately targeted to membranes. Then, even if the membrane localization accuracy is such that it is unclear which side a barcode is on, the barcodes on a membrane could still be used to establish a pair of neurons that contacts this boundary. This use of barcodes for constraining neuron pairs at boundaries would lead to a distinct algorithmic framework from the preliminary one demonstrated here, where barcodes are used to guide supervoxel mergers within a BPM that is established without using barcode information. Alternatively, an approach that “flood-fills” (Januszewski et al., 2016) outward from identified barcodes would not require barcodes to be present in every subcellular compartment, instead merely limiting the size of the volume over which accurate flood-filling would be necessary. This might allow far fewer barcodes to be used, yet still provide a benefit by making any segmentation errors into local perturbations that do not propagate over long distances, greatly limiting the impact of local errors on attributions of global cell shape. Thus, different algorithmic frameworks place different requirements on the spatial distributions of barcodes, and on the ways in which barcodes benefit the pipeline, and often, these requirements are more favorable than in the pessimistic baseline case studied here.

Previous work has shown neuronal barcoding to provide information relevant to identifying long-range projections: one can identify a process purely from a barcode located distally, without having to trace it all the way from the parent cell body via image analysis (Kebschull et al., 2016). The use of digital barcodes converts segmentation from a problem in which only local information is available, and hence in which errors in neuronal tracing accumulate with the total distance traced along an axon or dendrite, to a problem in which global information is available, and hence it is only necessary to trace an axon over a short distance from one barcode to another. What is less clear is to what extent barcodes can also assist with the local dense reconstruction problem, i.e., aid the accuracy with which a small volume can be densely reconstructed. We have here through simulations shown that barcodes can also aid in the local reconstruction problem, if the barcodes can be expressed in the cell at a sufficiently high density.

While our analysis was based on current forms of ExM, we expect ExM to continue to improve and make optical brain mapping even more feasible. A higher expansion factor—which may be achieved through additional iterations of expansion on the sample (Chang et al., 2017)—if it results in better resolution, may improve the reconstruction accuracy of thin processes. The resolution of double expansion is ~25 nm, but the error due to the gelation and expansion chemistry is ~5–10 nm (Chang et al., 2017), suggesting that higher expansion factors could, in principle, reveal yet more biological information. Improving the localization accuracy of the fluorescent probes themselves will become increasingly important with higher expansion factors, as it imposes an upper limit on the effective resolution. For example, small molecule fluorophores could be devised which tile the cell membrane more densely than expressed fluorescent proteins as used here. In this way, we would achieve a dense, spatially precise labeling of cell membrane boundaries, with higher localization accuracy than we have pessimistically assumed here for the case of secondary-antibody-tagged membrane-targeted fluorescent proteins (e.g., <5 vs. ~20 nm). Moreover, while we have assumed the use of standard confocal microscopes here, note that more isotropic, compact point-spread functions can be obtained in optical microscopy, which would improve resolution (Gustafsson et al., 1999; Shao et al., 2008; Chen et al., 2014). All of these approaches are in principle compatible with ExM.

In summary, we have demonstrated, via simulations of a conservative baseline case, that dense optical 3-D neural morphology reconstruction using ExM may be feasible, and have illustrated preliminary examples of algorithmic approaches that can be used to implement such an approach, both with and without digital barcodes. This should be viewed as the beginning of a much broader exploration of a vast design space for optical and molecularly annotated connectomics that is enabled by ExM, *in-situ* molecular multiplexing, machine learning, and other emerging techniques.

## Materials and methods

### Simulation of expansion microscopy images

We implemented the simulator in MATLAB R2016A and it was run on a workstation with an NVIDIA K40c GPU and 128GB of RAM. First, the input volume was re-sampled with 6 nm of isotropic resolution and the voxels that correspond to the membrane of each neuron were identified by detecting the boundary of each segment. In order to reflect the finite localization accuracy in ExM (which originates from non-zero mesh size of the hydrogel and the non-zero size of antibodies used for tagging specific proteins Chang et al., 2017), fluorophores were assigned with random labeling densities near the boundary voxels with a standard deviation of 20 nm. Fluorophores were also added to non-membrane parts of the volume, with random densities, to simulate fluorophores with non-specific binding and auto-fluorescence. Then, local clusters of fluorophores, or puncta, were created by convolving each fluorophore with a random size Gaussian kernel to reflect local clusters of fluorophores which result from multiple fluorophores being present on a single antibody-delivered tag, and from multiple antibodies binding to a single fluorescent protein target. The synthetic volume was projected onto a 3-D image stack by convolving the volume with the 3-D point-spread-function (PSF) of a confocal microscope with an objective lens with 40x magnification and 1.15 numerical aperture. The PSF was obtained by combining the excitation PSF and the emission PSF, which were in turn computed by evaluating the following Debye integral (Gu, 2000) where k, λ, v, u, α, P(θ), *J*_0_(·) denote the wave number, the wavelength, normalized radial coordinate, normalized axial coordinate, the half-angle of the numerical aperture, the apodization function of the microscope and the zeroth order Bessel function of the first kind, respectively:

(3) $$\begin{array}{r} U\left(v,u\right)=\frac{2\pi i}{\lambda }e^{-ikz}\int_{0}^{\alpha }P\left(\theta \right)J_{0}\left(\frac{v\text{ sin }\theta }{\text{sin }\alpha }\right)\exp\left(\frac{iu\text{ sin}{\;}^{2}\frac{\theta }{2}}{2\text{ sin}{\;}^{2}\frac{\alpha }{2}}\right)\text{sin }\theta \;d\theta . \end{array}$$

For modeling 20x ExM, the PSF was scaled down by a factor of 20 instead of expanding the volume by a factor of 20, for efficient computation. Finally, Poisson noise and read-out noise were added to the image (Supplementary Figure 1). Almost every parameter (e.g., labeling density, noise level) in each step of the simulation was randomized to reflect the stochastic nature of fluorescent labeling and imaging and to avoid over-fitting our analysis to a specific imaging condition. For simplicity, it was assumed that barcodes were randomly distributed over the volume, including throughout fine processes, and also that they can be read out without error. Table 1 summarizes on the simulation parameters. All parameters are in units corresponding to the original scale of the tissues (i.e., before expansion). The confocal microscopy parameters were matched to values of a typical confocal microscope, and the fluorophore density parameters were chosen to match the qualitative appearance of the simulated images to that of actual 20x ExM images (Chang et al., 2017). Localization accuracy of 20 nm was assumed, as our current 20x ExM protocol uses both primary and secondary antibodies to deliver the fluorescent label, each of which has a size around 14 nm (i.e., if we assume that two localization error vectors are uncorrelated, the added localization accuracy can be calculated as $\sqrt{{\left(14\;nm\right)}^{2}+{\left(14\;nm\right)}^{2\;}}≅\;20\;nm$).

Four different ground truth volumes (Supplementary Figure 2) were used for the simulation and the size of each volume was ~3 μm in each dimension. Twelve images from three ground truth volumes were used for training the segmentation algorithm and one image from a different ground truth volume was used for testing the algorithm. The simulated images from a single ground truth volume were significantly different as the simulation involved randomized fluorophore allocation as well as many randomized parameters, and hence, we took advantage of this as a part of the data augmentation for training the algorithms.

### Boundary detection

We implemented a ConvNet for boundary detection (Ciresan et al., 2012) using MATLAB R2016A with MatConvNet (Sun, 2015; Vedaldi and Lenc, 2015) and the computation was performed on a workstation with a NVidia K40c GPU and a medium size cluster with multiple GPUs. To train this network, we first implemented a 2-D ConvNet that had the same architecture except that it had only 2-D convolution layers (i.e., the filter size in 11th, 12th, and 13th convolution layers was 3 × 3 × 1 instead of 3 × 3 × 3), and trained this network. Then, the weights in the first to eighth convolution layers were taken to initialize the same part of the 3-D ConvNet. Then, the 3-D ConvNet was trained on a patch-by-patch basis with back-propagation and stochastic gradient descent with a learning rate of 0.001, a momentum of 0.9 and a batch size of 100. The patches were randomly sampled from 12 training volumes. In order to deal with the training imbalance (i.e., there are more non-boundary pixels than boundary pixels), the input patches were sampled to match their numbers. Data augmentation was done by flipping and rotating the input patches. In order to exploit as much 3-D information as possible to further improve the accuracy of the BPM, two ConvNets with the same architecture were trained with two different input orientations—one in xy (85 × 85 × 7) and the other in xz/yz (85 × 7 × 85, 7 × 85 × 85).

For inference (i.e., obtaining the BPM from the image), we implemented a forward pass function that can be applied to a volume of arbitrary size instead of using a sliding window for patch-by-patch evaluation, similar to ZNN (Zlateski et al., 2016), to reduce the number of redundant convolution operations for speed up. Using two ConvNets, three BPMs were obtained for a single volume, and then the median value of three was taken for each voxel to obtain the final BPM (Supplementary Figure 3).

### Over-segmentation and agglomeration

We applied a 3-D median filter to the BPM to make the boundary smooth and an H-minima transform to remove local minima that were too shallow with a threshold value *T* of 0.01 as pre-processing steps followed by watershed transformation to obtain the initial over-segmentation. Once over-segmentation was performed with a watershed transform, the segments were merged using GALA. To train GALA, the watershed segmentation result, the BPM and the input image were fed in and moment and histogram were chosen as the training features. The data sets used for training the ConvNets were used for training GALA as well. After training, we applied the algorithm to the test image with nine threshold values ranging from 0.1 to 0.9 to obtain a hierarchical segmentation tree. This tree was combined with the barcodes by assigning the barcodes to the segments, or nodes, at the lowest level of the segmentation tree. Barcodes that were within 30 nm from the boundary of segments were ignored, as the barcodes that are near a boundary might be assigned to neurons on the other side due to the finite localization accuracy of ExM assumed here and the potential for boundary shifts in the ConvNet output. After assigning the barcodes to the nodes at the lowest level, the numbers of distinct barcodes in the nodes at a higher level were examined. If the number of different barcodes of a node did not exceed one, all child nodes of the node were merged. This process was repeated until the top level of the tree was reached and the nodes with only 0 or 1 barcodes at the highest possible levels were identified. Finally, the nodes that had the same barcode were merged.

## Author contributions

YY and PD designed the segmentation algorithm. YY and JW built the ExM simulator. JC experimentally characterized ExM. AM and EB conceived of and led the project. YY, AM, and EB wrote the manuscript.

### Conflict of interest statement

The authors declare that the research was conducted in the absence of any commercial or financial relationships that could be construed as a potential conflict of interest.
