# Supplementary material for: Feasibility of 3D Reconstruction of Neural Morphology Using Expansion Microscopy and Barcode-Guided Agglomeration
Source: Front Comput Neurosci. 2017 Oct 24;11:97. doi: 10.3389/fncom.2017.00097 (PMC5660712; doi:10.3389/fncom.2017.00097)
Supplement: Supplementary file 1 [file Image1.pdf]

Supplementary Material

Feasibility of 3D Reconstruction of Neural Morphology using Expansion Microscopy and Barcode-Guided Agglomeration

Young-Gyu Yoon[1,2], Peilun Dai[2,3], Jeremy Wohlwend[1,2], Jae-Byum Chang[2,4], Adam H. Marblestone[2*] and Edward S. Boyden[2,3,5,6*]

*** Correspondence:**
Adam H. Marblestone and Edward S. Boyden
amarbles@mit.edu, esb@media.mit.edu

1 Supplementary Data

1.1 Supplementary Figures

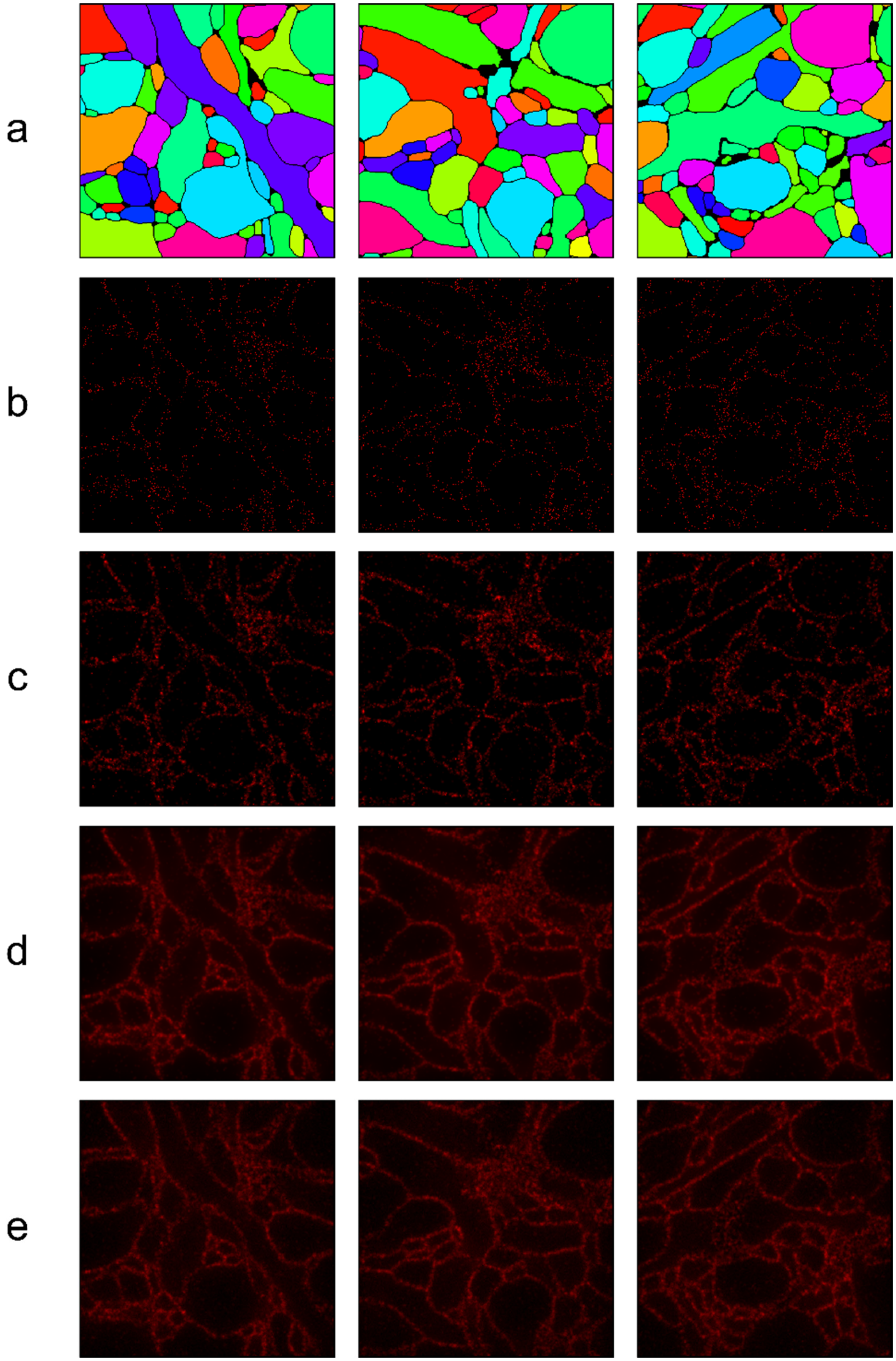
a
b
c
d
e

Supplementary Figure 1. Simulation procedure. (a) Ground truth input dataset obtained from a fully reconstructed electron microscopic volume. (b) Fluorophores were randomly assigned. (c) Local clusters of fluorophores, or puncta, were created by convolving each fluorophore with a random size Gaussian kernel to reflect local clusters of multiple fluorophores on antibodies bound to protein targets. (d) The synthetic volume was projected to a 3-D image stack by convolving the volume with the 3-D point-spread-function (PSF) of a confocal microscope. (e) Poisson noise and read-out noise were added to the image.

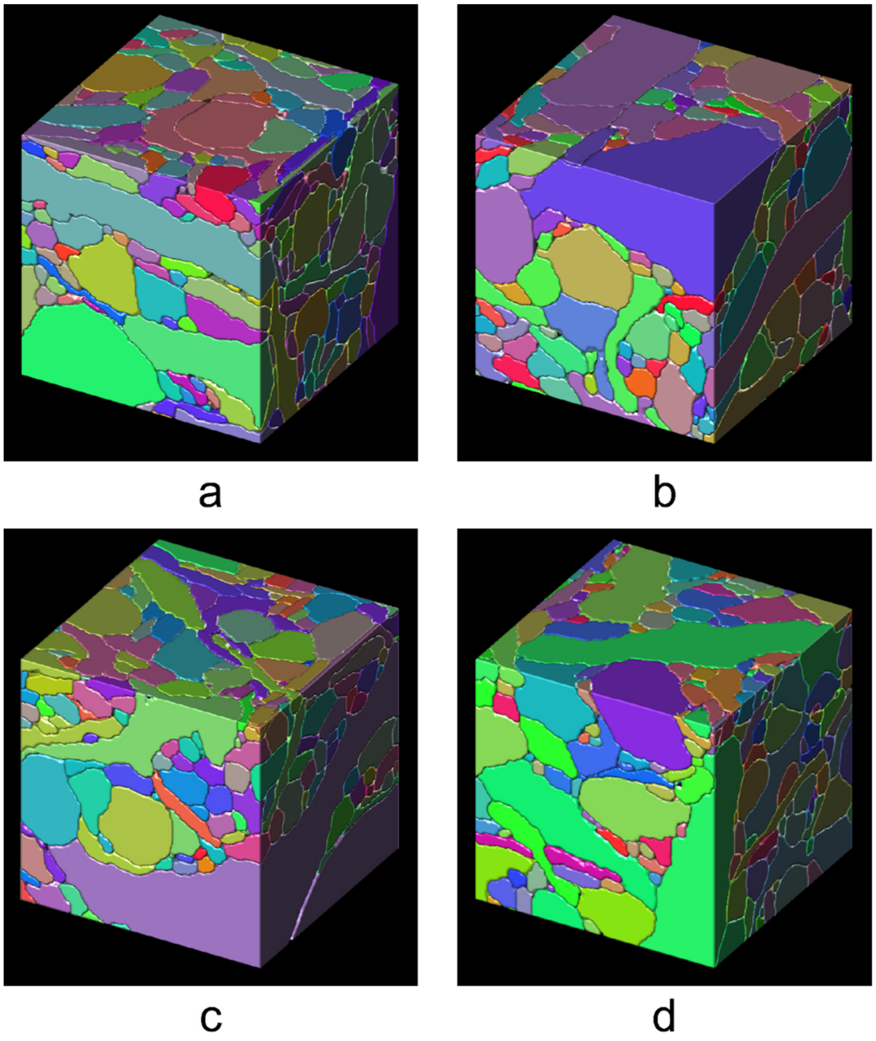

Supplementary Figure 2. 3-D surface renderings of the simulation input data sets. (a-c) 3-D renderings of the ground truth input data sets used for the simulation of ExM images for training the segmentation algorithm. (d) 3-D rendering of the ground truth input data set used for the simulation of an ExM image for testing the segmentation algorithm.

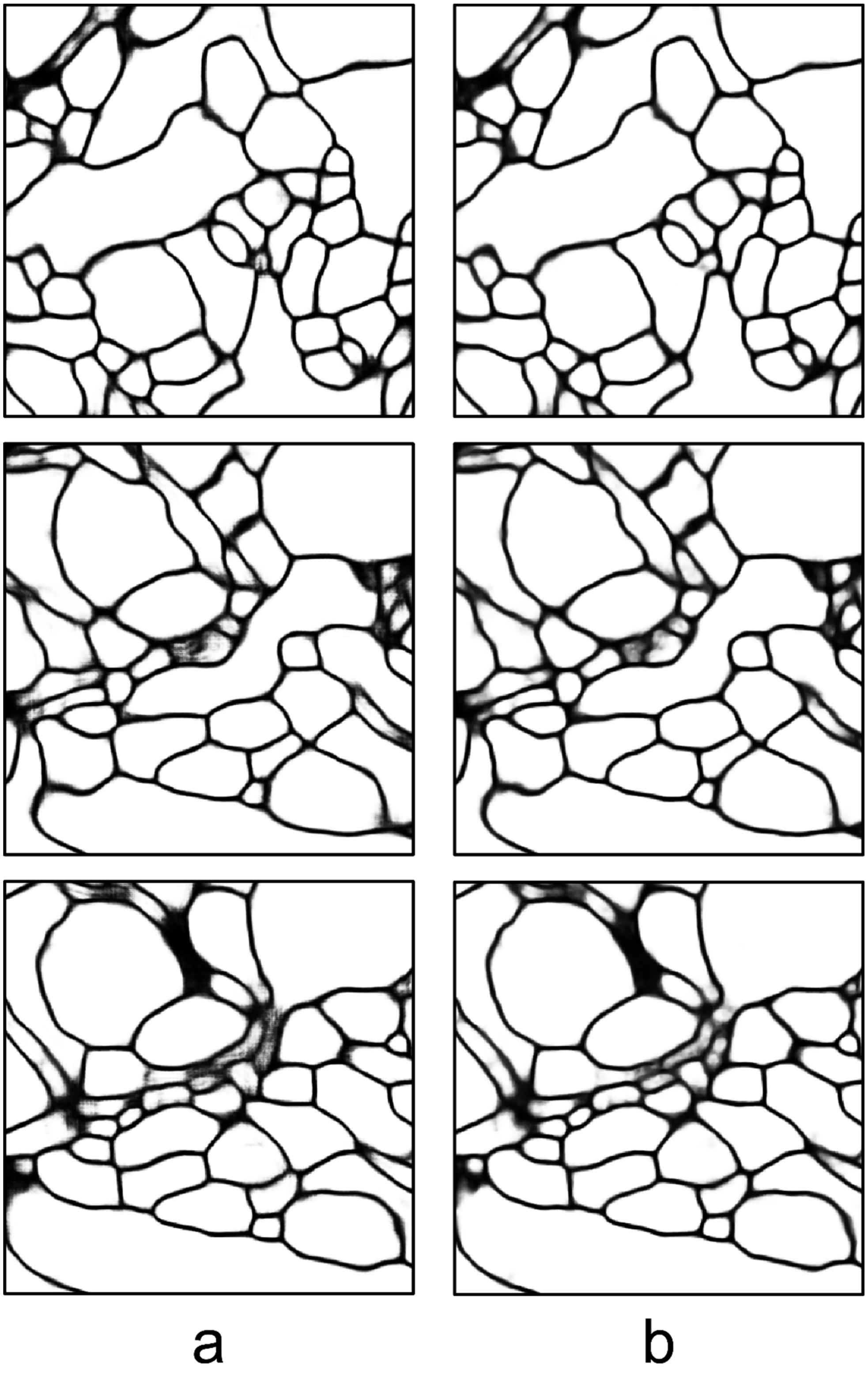

Supplementary Figure 3. Boundary probability map before and after post-processing. (a) Boundary probability map straight out of a ConvNet. (b) The boundary probability map was post-processed by taking the median value of three ConvNet outputs and applying a 3-D median filter.
